# Genetic analysis of an Indian family with members affected with Waardenburg syndrome and Duchenne muscular dystrophy

**Published:** 2012-07-20

**Authors:** Saketh Kapoor, Parayil Sankaran Bindu, Arun B. Taly, Sanjib Sinha, Narayanappa Gayathri, S. Vasantha Rani, Giriraj Ratan Chandak, Arun Kumar

**Affiliations:** 1 Department of Molecular Reproduction, Development and Genetics, Indian Institute of Science, Bangalore, India; 2 Department of Neurology, National Institute of Mental Health and Neuro Sciences, Bangalore, India; 3 Department of Neuropathology, National Institute of Mental Health and Neuro Sciences, Bangalore, India; 4 Centre for Cellular and Molecular Biology, Hyderabad, India

## Abstract

**Purpose:**

Waardenburg syndrome (WS) is characterized by sensorineural hearing loss and pigmentation defects of the eye, skin, and hair. It is caused by mutations in one of the following genes: *PAX3* (paired box 3), *MITF* (microphthalmia-associated transcription factor), *EDNRB* (endothelin receptor type B), *EDN3* (endothelin 3), *SNAI2* (snail homolog 2, *Drosophila*) and *SOX10* (SRY-box containing gene 10). Duchenne muscular dystrophy (DMD) is an X-linked recessive disorder caused by mutations in the *DMD* gene. The purpose of this study was to identify the genetic causes of WS and DMD in an Indian family with two patients: one affected with WS and DMD, and another one affected with only WS.

**Methods:**

Blood samples were collected from individuals for genomic DNA isolation. To determine the linkage of this family to the eight known WS loci, microsatellite markers were selected from the candidate regions and used to genotype the family. Exon-specific intronic primers for *EDN3* were used to amplify and sequence DNA samples from affected individuals to detect mutations. A mutation in *DMD* was identified by multiplex PCR and multiplex ligation-dependent probe amplification method using exon-specific probes.

**Results:**

Pedigree analysis suggested segregation of WS as an autosomal recessive trait in the family. Haplotype analysis suggested linkage of the family to the WS4B (*EDN3*) locus. DNA sequencing identified a novel missense mutation p.T98M in *EDN3*. A deletion mutation was identified in *DMD*.

**Conclusions:**

This study reports a novel missense mutation in *EDN3* and a deletion mutation in *DMD* in the same Indian family. The present study will be helpful in genetic diagnosis of this family and increases the mutation spectrum of *EDN3*.

## Introduction

Waardenburg syndrome (WS) is a hereditary auditory-pigmentary syndrome characterized by pigmentary abnormalities of the hair, including a white forelock and premature graying, pigmentary changes of the iris such as heterochromia irides and brilliant blue eyes, lateral displacement of the medial canthi, and lacrimal points, congenital deafness, and intestinal abnormalities [1]. The association of hearing loss, pigmentary abnormalities, and intestinal malformation results from an abnormal proliferation, survival, migration, or differentiation of neural crest derived melanocytes of skin and inner ear, glia, and neurons of the peripheral and enteric nervous system [2]. The incidence of WS is around 1 in 42,000 in the general population. However, it occurs in 5%–6% of deaf individuals and is considered to be the most common autosomal-dominant form of syndromic deafness [3]. It is clinically and genetically heterogeneous and is classified into four types (viz., type I to IV) based on the presence of variable clinical characteristics and additional symptoms. WS type I is characterized by the presence of dystopia canthorum (lateral displacement of inner canthi). WS type II is distinguished from type I by the absence of dystopia canthorum. WS type III patients have limb hypoplasia in addition to dystopia canthorum. WS type IV has an additional feature of Hirschsprung disease [2]. Genetic analysis has identified at least eight loci for WS: WS1/WS3 on chromosome 2q36.1, WS2A on chromosome 3p14.1-p12.3, WS2B on chromosome 1p21-p13.3, WS2C on chromosome 8p23, WS2D on chromosome 8q11.21, WS2E/WS4C on chromosome 22q13.1, WS4A on chromosome 13q22.3, and WS4B on chromosome 20q13.2-q13.3 (OMIM). The genes for six loci are known: WS1/WS3-*PAX3* (paired box 3), WS2A-*MITF* (micropthalmia-associated transcription factor), WS2D-*SNAI2* (snail homolog 2, *Drosophila*), WS2E/WS4C-*SOX10* (SRY-box containing gene 10), WS4A-*EDNRB* (endothelin receptor type B) and WS4B-*EDN3* (endothelin 3) [4-12].

Duchenne muscular dystrophy (DMD) is the most common X-linked recessive disease. It is estimated to affect 1 in 3,500 newborn males worldwide [13] and caused by mutations in the *DMD* gene, encoding a cytoskeletal protein dystrophin. *DMD* is the largest human gene that spans >2,200 kb of DNA and is composed of 79 exons [14]. Deletions account for approximately 65% of DMD mutations, duplications occur in approximately 6%–10% of cases and the remaining 30%–35% of mutations consist of small deletions, insertions, point mutations or splicing mutations, most of which introduce a premature stop codon [14-23].

Here we report on the genetic analysis of a consanguineous family from the south Indian state of Karnataka with members affected with two disorders: Waardenburg syndrome and Duchenne muscular dystrophy.

## Methods

### Patients

**Figure 1:**
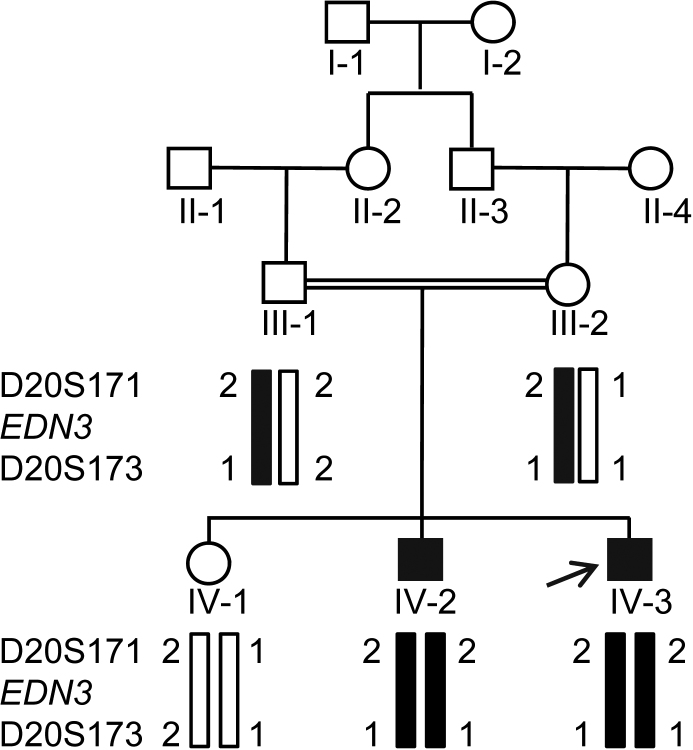
The haplotype analysis of the family with microsatellite markers from the *EDN3* candidate region. The disease haplotype 2–1 is shown by black bars. Note the affected individuals (IV-2 and IV-3) are homozygous for the disease haplotype, whereas both parents III- 1 and III-2 are heterozygous for the disease haplotype and are therefore carriers for the mutation. An arrow marks the index case.

We ascertained a consanguineous family (Figure 1) with one individual (IV-3) affected with WS and DMD, and the other one (IV-2) affected with only WS from a south Indian state of Karnataka in the Department of Neurology, National Institute of Mental Health and Neuro Sciences, Bangalore. Both affected individuals were examined by Parayil Sankaran Bindu, Arun B. Taly and Sanjib Sinha. A detailed description of their clinical symptoms is given below.

#### Individual IV-3

**Figure 2:**
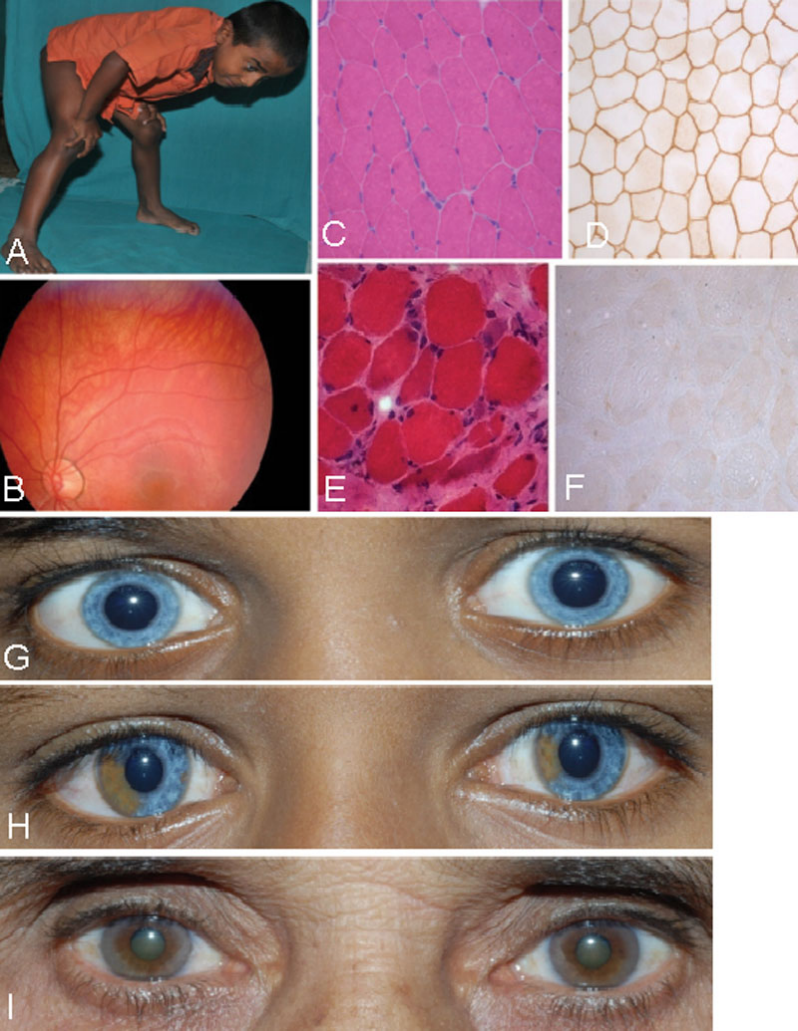
Clinical features of the affected individuals. **A**: Photograph of affected individual IV-3. Note the classical Gower’s sign on trying to get up from the sitting position. **B**: Fundus photograph of affected individual IV-3. Note the depigmented retina and underlying choroid vessels. **C**-**F**: Transverse sections of skeletal muscle: **C** and **D** from an unrelated normal individual and **E** and **F** from affected individual IV-3. Note normal polygonal myofibers with peripheral nuclei and uniform diameter in panel **C** (hematoxylin and eosin staining) and normal positive immunostaining of DMD protein along the sarcolemma in all the fibers in panel **D**. Note rounding, variation in diameter, central nuclei, regenerating fibers and fibrosis in panel **E** (hematoxylin and eosin staining), and total absence of DMD staining in all the fibers in panel **F**. **G**: Partial facial photograph of affected individual IV-3 showing blue iris. **H**: Partial facial photograph of affected individual IV-2 showing heterochromia of iris. **I**: Partial facial photograph of paternal grandmother II-2 showing heterochromia of iris.

The propositus, a 9-year-old boy, was the third child born to consanguineous parents after an uneventful pregnancy and delivery. His developmental milestones were normal except for speech delay. A formal evaluation for the speech delay at the age of three years revealed profound bilateral sensorineural hearing loss. Since the age of six years, he developed gradually progressive difficulty in walking and getting up from sitting position. He had blue iris, faint white forelock, confluent eyebrows, bilateral calf hypertrophy, mild tendon Achilles contracture, exaggerated lumbar lordosis, and a waddling gait. He had symmetric weakness of proximal muscles of all four limbs and truncal muscles. All stretch reflexes were sluggish except for the ankle jerks. Gower’s sign was evident on getting up from the sitting position (Figure 2A). Ophthalmological evaluation showed retina with visible choroidal vessels and depigmented iridis (Figure 2B,G). Waardenburg index was 0.572, thus ruling out dystopia canthorum. Laboratory evaluation showed normal hemogram and routine biochemical parameters. Serum creatine phosphokinase was 3,218 IU/l (n<170 IU/l). Nerve conduction velocity studies that involved right median, ulnar and common peroneal, and sural nerve were normal. Electromyography of right biceps and vastus lateralis revealed a myopathic pattern. Audiometric evaluation showed profound sensorineural hearing loss and absence of bilateral brainstem auditory evoked responses. Magnetic resonance imaging of the brain showed normal myelination pattern. A left quadriceps biopsy revealed marked variation in fiber diameter with evidence of internal nuclei, occasional myophagocytosis, regenerating fiber and splitting, suggestive of muscular dystrophy. On immunohistochemistry with an anti-dystrophin antibody (Novocastra Laboratories, UK), there was absence of staining of dystrophin (Figure 2F). The above clinical symptoms suggested that this patient has both WS and DMD.

#### Individual IV-2

The 11-year-old elder brother of the index patient had a history of Hirschsprung disease that required surgery at the age of 3 months. He had iris discoloration at birth but did not report any hearing problem till the age of 8 years. On examination, he had heterochromia of iris (Figure 2H) and a normal neurologic examination. Ophthalmological evaluation revealed hypopigmented iris and choroid with visualization of the choroidal vessels. Waardenburg index was 0.4059, thus ruling out dystopia canthorum. Audiometry revealed profound hearing loss on left side with absence of auditory brainstem evoked responses. Laboratory evaluation showed normal creatine kinase levels. Nerve conduction velocity studies and magnetic resonance imaging of the brain were normal. The above clinical symptoms suggested that he has only WS, and it could be WS type IV (WS4).

A detailed clinical examination of their parents, elder sister and paternal grandmother showed heterochromic iris only in the grandmother II-2 (Figure 2I).

### Mutation analysis

Following informed consent, three-five milliliter of peripheral blood sample was collected in a Vacutainer EDTA^TM^ tube (Beckton-Dickinson, Franklin Lakes, NJ) from each individual for genomic DNA isolation using a Wizard^®^ Genomic DNA Extraction Kit (Promega, Madison, WI). This research followed the tenets of the Declaration of Helsinki and the guidelines of the Indian Council of Medical Research, New Delhi. Although the clinical features in the affected individual IV-2 suggested this family to be of WS type IV, we still went ahead and selected two microsatellite markers from each of the eight WS loci (Table 1) and used them to genotype the family, according to Kumar et al. [24].

**Table 1:** Microsatellite markers from the candidate regions of eight known WS Loci.

**Locus**	**Chromosome location**	**Gene**	Marker*
WS1/WS3	2q36.1	*PAX3*	D2S2197, D2S2300
WS2A	3p14.1-p12.3	*MITF*	D3S1296, D3S1566
WS2B	1p21-p13.3		D1S495, D1S248
WS2C	8p23		D8S561, D8S1819
WS2D	8q11.21	*SNAI2*	D8S1716, D8S1745
WS2E/WS4C	22q13.1	*SOX10*	D22S1045, D22S1156
WS4A	13q22.3	*EDNRB*	D13S1281, D13S160
WS4B	20q13.2-q13.3	*EDN3*	D20S171, D20S173

*Marker order was established using the sequence map from the UCSC Genome Bioinformatics site.

For mutational analysis, the entire coding region of the *EDN3* gene (GenBank NM_000114.2) was amplified using primers that amplify all coding exons and their intron-exon junctions (Table 2). Primers were designed using the gene sequence from the UCSC Genome Bioinformatics site. Sequences and PCR conditions of these primers are provided in Table 2. Mutations were identified by sequencing the PCR products from an affected individual from the family on an ABIprism A370-automated sequencer (PE Biosystems, Foster City, CA). Once a mutation was identified, all members of the family were examined for the presence of the mutation by sequencing.

**Table 2:** Details of PCR primers used in mutation analysis of the *EDN3* gene.

**Exon**	**Primer**	**Primer sequence (5′ to 3′)**	**Tm (°C)**	**Amplicon size (bp)**
1*	EN1bF	F:gaaaagcccgagccacagccggc	64	379
** **	EN1bR	R:ccgcgacgcacatcttctccgcg	** **	** **
2	EN2F	F:cagacattttgcttgctccacc	62	528
** **	EN2R	R:caggctctgggctaactgagc	** **	** **
3	EN3F	F:ggcggtggttctcgctccacac	56	372
** **	EN3R	R:caggatgtgactgaactatccta	** **	** **
4	EN4F	F:tggggaacgcactaatgtgctca	62	336
** **	EN4R	R:agaaacggtccaccaaaggcacc	** **	** **
5	EN5F	F:ttccagtctggtggtaggctcg	56	458
** **	EN5R	R:gtattgttaagtggggactctttg	** **	** **

Abbreviations: F, forward primer; R, reverse primer; bp, base pairs; and Tm, Annealing temperature. * Works with 5% DMSO.

The mutation analysis of the *DMD* gene was performed using multiplex PCR and multiplex ligation-dependent probe amplification (MLPA) technique and a kit from MRC-Holland, Amsterdam, the Netherlands according to the manufacturer’s recommendations. The kit contains specific probes for each of the 79 exons of the *DMD* gene on Xp21.2 and thus allows analysis of deletion and/or duplication of one or more of these exons. Briefly, 200 ng DNA was denatured and hybridized overnight at 60 °C with the probe mix. Samples were then treated with Ligase 65 for 15 min at 54 °C. The reactions were stopped by incubation at 98 °C for 5 min. Finally, PCR amplification was performed with the specific SALSA FAM PCR primers. Amplification products were run on an ABI PRISM 3730 Genetic Analyzer (PE Biosystems, Foster City, CA) and the obtained data were analyzed by using the Gene Marker 2.0 Software. Deletions of a probe’s recognition sequence on the X-chromosome will lead to a complete absence of the corresponding probe amplification product in males, whereas female heterozygotes are recognizable by a 35%–50% reduction in relative peak area.

## Results and Discussion

**Figure 3:**
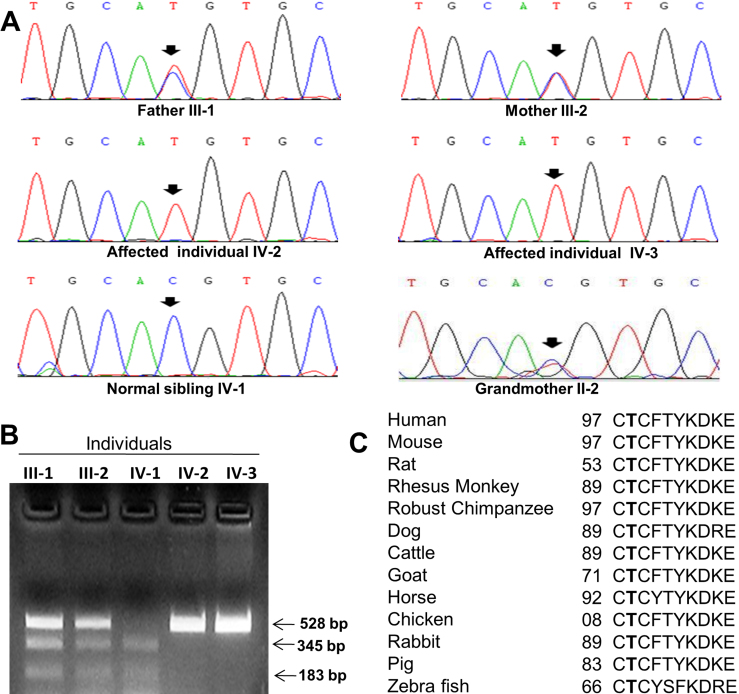
Mutation analysis of the *EDN3* gene in the family. **A**: Sequencing chromatograms of individuals from the family. Note the homozygous change C>T in both affected individuals IV-2 and IV-3 (marked by arrows). The normal sibling IV-1 is homozygous for the wild-type allele, whereas both parents (III-2 and III-3) and grandmother (II-2) are heterozygous for the change (see double peaks marked by arrows). **B**: RFLP analysis to show segregation of the mutation. Note affected individuals have only 528 bp fragment due to loss of the Eco72I site, the normal sibling has 345 and 183 bp fragments, and both carrier parents have all the three fragments. **C**: Conservation of the threonine (T) residue in different orthologs. The threonine residues are shown in bold letters. The number refers to the position of amino acid residue.

The visual inspection of the family suggested segregation of WS as an autosomal recessive trait (Figure 1). Haplotype analysis using microsatellite markers from each of the eight known WS loci suggested linkage of the family to the WS4B locus on chromosome 20q13.2-q13.3 (Figure 1 and Table 1), indicating that WS is caused by a mutation in the *EDN3* gene. The genotypes of individuals for microsatellite markers from the other seven WS loci are given in Table 3. To determine the exact nature of the mutation, the entire coding region of *EDN3* from the affected individual IV-2 was amplified using 5 sets of PCR primers (Table 2) and sequenced. The results revealed a novel homozygous substitution mutation, c.293C>T in exon 2, changing codon position 98 from threonine to methionine (p.T98M; Figure 3A). The affected sibling IV-3 was also homozygous for the mutation, both parents (III-1 and III-2) and paternal grandmother (II-2) were heterozygous and the normal sibling IV-1 was homozygous for the wild-type allele (Figure 3A). The 528 bp wild-type exon 2 amplicon contains a site for the restriction endonuclease Eco72I, cleaving it into two fragments of 345 bp and 183 bp. The mutation abolishes this restriction site, thus retaining the undigested 528 bp amplicon in affected indivuduals. We therefore developed a restriction fragment length polymorphism (RFLP) analysis to determine if the mutation was present in 50 normal controls. The analysis showed absence of the mutation in 50 ethnically matched controls, further suggesting that the c.293C>T change is a mutation (data not shown). The analysis also showed segregation of the mutation in the family (Figure 3B). In addition, the pathogenic nature of the mutation also comes from the fact that threonine residue at position 98 is conserved in different *EDN3* orthologs from human to zebra fish (Figure 3C). According to the Mutation Taster, the mutation is predicted to be disease causing with a p-value (probability) of 0.52. We then used two other in silico methods, PolyPhen-2 and SIFT, to see the effect of this mutation on the protein function. The effect of this mutation on EDN3 was predicted to be probably damaging by PolyPhen-2 with a score of 1 (score ranges from 0 to a positive number, where 0 is neutral, and a high positive number is damaging). SIFT analysis predicted this mutation to be damaging and intolerant with a score of zero (score ranges from 0 to 1, where 0 is damaging and 1 is neutral).

**Table 3:** Genotypes of individuals for markers from different WS loci.

** **	** **	**Genotype**				
**Locus**	**Marker**	**Individual III-1**	**Individual III-2**	**Individual IV-1**	**Individual IV-2**	**Individual IV-3**
WS1/WS3	D2S2197	1 2	1 2	1 1	1 1	2 2
** **	D2S2300	1 1	1 2	1 1	1 1	1 2
WS2A	D3S1296	2 3	1 2	2 3	1 3	1 2
** **	D3S1566	3 4	1 2	1 3	2 3	2 4
WS2B	D1S495	1 3	2 4	1 2	2 3	1 4
** **	D1S248	1 2	2 2	2 2	1 2	2 2
WS2C	D8S561	1 1	1 2	1 1	1 1	1 2
** **	D8S1819	1 1	2 3	1 2	1 2	1 3
WS2D	D8S1716	2 2	1 3	1 2	2 3	1 2
** **	D8S1745	1 2	1 2	1 2	1 1	2 2
WS2E/WS4C	D22S1045	2 3	1 2	1 2	1 2	1 2
** **	D22S1156	2 3	1 3	2 3	2 3	2 3
WS4A	D13S1281	2 2	1 2	1 2	1 2	1 2
** **	D13S160	1 2	2 2	1 2	1 2	1 2

**Figure 4:**
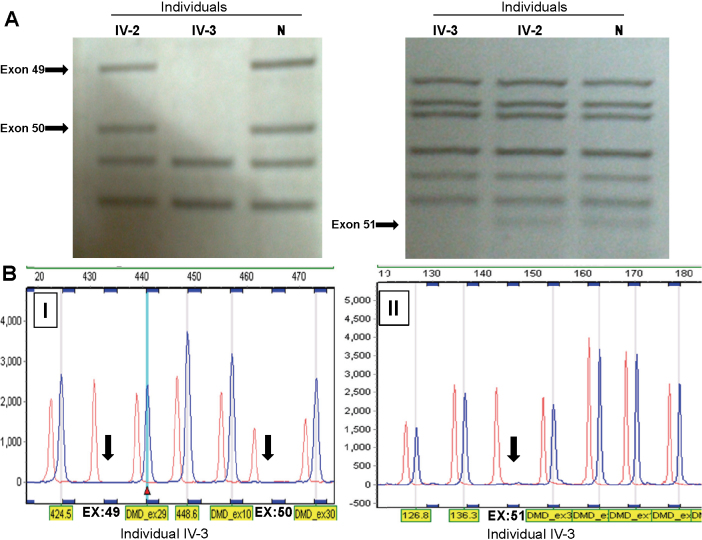
Deletion analysis of the *DMD* gene. **A**: Results of multiplex PCR showing the deletion of exons 49, 50 and 51 (marked by arrows) in affected individual IV-3. **B**: MLPA analysis of the affected individual IV-3 showing the deletion of exons 49, 50 and 51 (marked by arrows). Abbreviation: N, normal unrelated individual.

Multiplex PCR and MLPA analyses of the affected individual IV-3 showed that he has a deletion from exons 49–51 in the *DMD* gene (Figure 4). MLPA analysis showed absence of the deletion in his mother (III-2), suggesting that it is a de novo mutation (data not shown). His sister (IV-1) also did not have this mutation (data not shown). This deletion causes a complete absence of the DMD protein in muscle tissues (Figure 2F) of the affected individual, and thus causing the phenotype.

A total of 10 mutations have been reported so far in the *EDN3* gene in WS patients of different ethnic origins [2,9,25-30]. The novel mutation p.T98M reported here, along with two other mutations p.H112R and p.T98K, affect one of the 21 amino acids that constitute active peptide from amino acid positions 97–117 in endothelin-3 [2,29]. Further, with the mutation reported here, the total number of mutations in the *EDN3* gene is 11 (Table 4).

**Table 4:** Mutations reported so far in the *EDN3* gene.

**Sl. #**	**Mutation**	**Exon**	**Nature of mutation**	**State of zygosity**	**Effect on protein**	**Ethnic origin of family**	**Reference**
1	c.163G>T (p.E55X)	2	Nonsense	Homozygous	Predicted to result in complete absence of active form of peptide	French	[26]
2	c.262_263delGCinsT (p.Ala88SerfsX12)	2	Frameshift	Homozygous	Predicted to result in complete absence of active form of peptide	1 French & 1 Bosnian	[2,9]
3	c.277C>G (p.R93G)	2	Missense	Homozygous	Predicted to impair furin-mediated proteolytic cleavage of preproendothelin	Egyptian	[30]
4	c.286C>T (p.R96C)	2	Missense	Homozygous	Predicted to impair furin-mediated proteolytic cleavage of preproendothelin	French	[2]
5	c.293C>A (p.T98K)	2	Missense	Homozygous	Predicted to result in absence of active form of protein	Indian	[2]
6	c.293C>T (p.T98M)	2	Missense	Homozygous	Predicted to result in absence of active form of protein	Indian	Present study
7	c.335A>G (p.H112R)	2	Missense	Homozygous	Could disrupt the formation of disulphide bond	Spanish	[29]
8	c.380A>G (p.Y127C)	3	Missense	Homozygous	Could alter disulphide bond formation in the endothelin and/or the ETlike peptide regions	Indian	[28]
9	c.476G>T (p.C159F)	3	Missense	Homozygous	Presumably results in less efficient cleavage or even complete failure of cleavage of prepro-endothelin	3 Pakistani	[2,25]
10	c.507C>A (p.C169X)	3	Nonsense	Heterozygous	Prevents the disulphide bond formation and probably generates an inappropriately cleaved, inactive proendothelin	Yugoslavian	[27]
11	c.517T>C (p.C173R)	3	Missense	Compound heterozygosity with p.T98K	Prevents the disulphide bond formation	Indian	[2]

Endothelins (EDNs) are a family of three active peptides EDN1, END2, and EDN3 that act as ligands and recognized by two G-protein coupled heptahelical receptors known as endothelin receptors, EDNRA and EDNRB. EDN3 preferentially binds the EDNRB [31]. EDN3 is produced as preproendothelin-3 encoded by the *EDN3* gene. The biologically active 21 amino acid long EDN3 is produced by proteolytic cleavage of preproendothelin-3 by endopeptidases to yield proendothelin-3, which is finally cleaved by endothelin converting enzyme-1 to produce the mature active EDN3 [32,33]. Studies have shown that EDN3/EDNRB interaction is required for the proper development of neural crest derived melanocytes and enteric neurons [34-36]. Mouse models for homozygous mutations in the *EDNRB* or *EDN3* genes show pigmentation anomaly, aganglionic megacolon and cochlear disorder [31,37]. We suggest that the disease phenotype in the present family could be due to the absence of the active form of the protein as the mutation p.T98M might impair the EDN3 activity. Although both parents (III-1 and III-2) and grandmother (II-2) are heterozygous for the mutation (Figure 3), only the grandmother showed heterochromic iris (Figure 2I). However, it is not completely unexpected as heterozygous individuals for the *EDN3* mutations have been reported to show a few clinical features of WS [25,29]. The manifestation of a few of the WS clinical symptoms in heterozygous individuals could be due to environmental factors, multigenic inheritance, modifier genes or stochastic events on cell fate or cell differentiation in early embryogenesis [25,27].

In summary, we report an interesting Indian family with members affected with two different disorders, WS and DMD. The WS is caused by a novel missense mutation, whereas DMD resulted due to a deletion. The present information will be useful to provide rapid prenatal diagnosis and genetic counseling for WS to the family and their relatives using a PCR-RFLP method developed during the study.
